# Who are the haters? A corpus-based demographic analysis of authors of hate speech

**DOI:** 10.3389/frai.2023.986890

**Published:** 2023-05-19

**Authors:** Lisa Hilte, Ilia Markov, Nikola Ljubešić, Darja Fišer, Walter Daelemans

**Affiliations:** ^1^CLIPS, Department of Linguistics, Faculty of Arts, University of Antwerp, Antwerp, Belgium; ^2^CLTL, Department of Language, Literature and Communication, Faculty of Humanities, Vrije Universiteit Amsterdam, Amsterdam, Netherlands; ^3^Department of Knowledge Technologies, Institut Jožef Stefan (IJS), Ljubljana, Slovenia; ^4^Laboratory for Cognitive Modeling, Faculty of Computer and Information Science, University of Ljubljana, Ljubljana, Slovenia; ^5^Institute of Contemporary History, Ljubljana, Slovenia; ^6^Department of Translation, Faculty of Arts, University of Ljubljana, Ljubljana, Slovenia

**Keywords:** hate speech, demographics, age, gender, language area

## Abstract

**Introduction:**

We examine the profiles of hate speech authors in a multilingual dataset of Facebook reactions to news posts discussing topics related to migrants and the LGBT+ community. The included languages are English, Dutch, Slovenian, and Croatian.

**Methods:**

First, all utterances were manually annotated as hateful or acceptable speech. Next, we used binary logistic regression to inspect how the production of hateful comments is impacted by authors' profiles (i.e., their age, gender, and language).

**Results:**

Our results corroborate previous findings: in all four languages, men produce more hateful comments than women, and people produce more hate speech as they grow older. But our findings also add important nuance to previously attested tendencies: specific age and gender dynamics vary slightly in different languages or cultures, suggesting that distinct (e.g., socio-political) realities are at play.

**Discussion:**

Finally, we discuss why author demographics are important in the study of hate speech: the profiles of prototypical “haters” can be used for hate speech detection, for sensibilization on and for counter-initiatives to the spread of (online) hatred.

## 1. Introduction

Hate speech is commonly defined as verbal communication that disparages an individual or a group on the basis of characteristics such as ethnicity, nationality, gender identity, sexual orientation, religion, and culture (Nockleby, 2000). In the present paper, we will use “hate speech” as an umbrella term covering some other closely related phenomena such as online harassment and offensive language use. Note that an equivalent term from social sciences is “socially unacceptable discourse” (SUD), which encompasses various types of offensive language (Fišer et al., 2017).

These last years, the phenomenon of hate speech has steadily grown and has become increasingly problematic and visible. It is pervasive on popular social media platforms and especially in so-called echo chambers, i.e., more niche online platforms that may be used to spread and propagate hatred. According to Suler (2004), people may act out more intensely (including, e.g., more hateful) in an online setting than in person because of the “disinhibition effect.” This effect is influenced by multiple aspects of the online setting, such as anonymity and asynchronicity (Suler, 2004).

As a response to this growing phenomenon of online hate speech, a wide range of stakeholders wants to understand and combat it. This includes governmental organizations such as law enforcement and security agencies, that are concerned with the real-life impact of online hate speech and want to combat it in order to counter radicalization and to prevent hate crimes—see for instance Relia et al. (2019) for the relationship between discrimination on social media and hate crimes in the USA. Furthermore, social media platforms may want or be obliged to detect and moderate hate speech, in order to keep discussions constructive and to improve the “health” or non-toxicity of interactions on their platforms.

In the present paper, we do not investigate online hate speech itself (as, e.g., Waseem et al., 2017; Markov et al., 2021 do), but rather its authors. From a large and multilingual dataset (including English, Dutch, Slovenian, and Croatian) consisting of online comments, we extract the demographic attributes of the creators of online hateful content. We focus on three key sociodemographic variables: age, gender identity, and language (area). The results provide insight in which kind of people are more likely to post hateful online content, which can in turn lead to a deeper understanding of the phenomenon of hate speech (see also Section Theoretical framework below).

The paper is structured as follows. First, Section Theoretical framework presents an overview of related research. Next, in Section Materials and methods, the data collection and methodology are described. In Sections Results and Discussion, finally, we respectively report on and discuss the results of the analyses.

## 2. Theoretical framework

In this Section, we first offer an overview of related work that highlights the importance of author demographics in hate speech detection (Section Importance of author demographics in hate speech detection), the most frequent use case of author demographics in hate speech research. Next, we summarize previously attested correlations between people's sociodemographic profiles and their production of and attitudes toward hate speech (Section The sociodemographic profiles of hate speech authors).

### 2.1. Importance of author demographics in hate speech detection

One way to increase our understanding of the phenomenon of hate speech consists in gaining insight in the profiles of the “haters,” i.e., the hateful content creators (Vidgen and Derczynski, 2020). A data-driven approach to this research question concerns the inclusion of meta-information on the users who created the hateful content. For instance, various aspects of their sociodemographic profiles can be included, but also information on their online behavior and affiliations, and their relation to other users (i.e., community context) (Vidgen and Derczynski, 2020). Below, we describe several ways in which information on the profiles of hateful content creators can contribute to more robust hate speech detection systems as well as to better countering strategies to offensive discourse.

First of all, previous research demonstrates that when user information is added to models–in addition to textual features–it can boost the performance of hate speech classification. For instance, when Waseem and Hovy (2016) combined textual features with users' gender identity on a dataset of tweets, classification performance improved slightly. And Qian et al. (2018) first modeled each user (based on their previous posts and on semantically similar posts by other users) in order to better understand their linguistic and behavioral patterns. This yielded an increase in classifier performance too. A final example that we will include here, is the study by Mishra et al. (2018), who incorporated “community-based” profiles of users based on properties of the authors' social network, arguing that users who are prone to posting hateful content tend to form (online) social groups. These profile features improved classification performance too.

Furthermore, insight in the profiles of hateful content creators can improve automated hate speech detection by addressing model bias. Sap et al. (2019) explored the role of racial (sociolect) bias in this respect. They found that when users' racial background (namely users being African-American or not) was provided to human annotators, the annotators were significantly less likely to label the messages as offensive, since they appeared aware that certain (potentially) offensive words or phrases can be used in non-offensive ways in African-American English. But when classification *models* were trained on the same dataset, the African-American English messages were labeled offensive twice as often, because the models did not account for this particular property of the dialect/sociolect. A similar systematic racial bias by a model was observed by Davidson et al. (2019): they too report messages written in African-American English to be automatically labeled as hateful at substantially higher rates. Huang et al. (2020) found evidence for demographic bias as well, showing how not only users' race or ethnicity led to biased classifiers, but also their age, nationality, and gender identity—three variables which largely correspond to the ones included in the present research design. Since text alone does not determine people's perception of offensiveness (see above), one could wonder whether it should be the sole source for models to base their decisions on. Hate speech detection models can benefit from the inclusion of confounding factors such as the social identity of content creators—which if not included, might lead to (e.g., demographically) biased classifiers.

Finally, user meta-information can aid the generation of counter-narrative: non-offensive responses to hate speech that provide argumentative feedback, which are considered an important strategy to fight online hatred and combat online radicalization (Schieb and Preuss, 2016; Chung et al., 2019). The profiling of “haters” can aid the development of effective and persuasive counter-narratives that are personalized or target-oriented, e.g., with respect to the user's demographic profile or online behavior. Chung et al. (2019), for instance, created a dataset consisting of pairs of hateful utterances and corresponding counter-narrative utterances, for which author gender, age, and educational level were taken into account. This meta-information enabled more accurate pairings and thus a more efficient countering of offensive discourse.

So the inclusion of meta-information on hateful content creators serves several purposes when automatically detecting hate speech. It can improve classification performance and gain insight in or even help to avoid different kinds of unintended biases. In addition, it can help the development of efficient counter-narratives. Our own analysis on the demographics of hateful content creators (Section Results) aims to complement the previous work addressed in this section, and to provide new insights in the profiles of prototypical online “haters” in terms of their age and gender identity, and this for several European language areas. In this paper, we want to provide an empirical basis to understand just *how* demography impacts people's production of hate speech.

### 2.2. The sociodemographic profiles of hate speech authors

Below we will describe previously attested correlations between people's profiles and their production of and attitudes toward hate speech. We will zoom in on two sociodemographic variables in particular, i.e., age and gender identity, as these variables are included in our own research design. Note that literature on this topic is very scarce and often limited to a specific platform, dataset, and language, and/or to a very specific type of hate speech. In addition, there do not yet seem to exist any studies on the impact of language (area) or culture (i.e., our third sociodemographic variable) on the production of hate speech.

With respect to age, De Smedt et al. (2018) found most authors of online jihadist hate speech on Twitter to be adults over 25 years old (95%). Only a small share were younger than 25 (5%). And the largest share of authors posting jihadist tweets were young adults between 20 and 35 years old. With respect to attitudes on and tolerance toward hate speech, Lambe (2004) found the following age pattern: the older a person was, the less willing they appeared to endorse censorship of hate speech, but not significantly so.

Regarding gender, Waseem and Hovy (2016) found that most authors (for whom the gender could be identified) in their dataset of hateful tweets were male. In their dataset of jihadist tweets, De Smedt et al. (2018) identified most perpetrators as men too (95%). As for people's attitudes on offensive language, women appear more likely than men to approve of censorship for hate speech (Lambe, 2004).

In Section Results, we will compare these previous findings to our own results with respect to the age and gender identity of hateful content creators in our dataset, and we will provide information on an additional sociodemographic variable: users' language or language area.

## 3. Materials and methods

Below, we discuss the dataset and data collection (Section Data and annotation), the sociodemographic variables included in the research design (Section Sociodemographic variables), and the method for the statistical analyses (Section Method).

### 3.1. Data and annotation

In order to create the dataset for the present research, we consulted the official Facebook pages of several mainstream media outlets in four languages: English, Dutch, Slovenian, and Croatian.^1^ On each of these Facebook pages, news articles that were published by the media outlets are (re-)published or (re-)shared as Facebook posts. Readers can leave written reactions to these posts and discuss the articles, resulting in a comment section. Our final corpus consists of a topic-based selection of posts and the related reader comments, with annotations (see below).

The specific media outlets were selected as follows: for each of the four languages, we chose the three media outlets that had the most-visited websites (according to the Alexa service)^2^ that also have popular Facebook pages. Table 1 offers an overview. While the entire variety of news content in a country is obviously not covered since our sample is not exhaustive, we are confident that the Facebook pages of the three most prominent news sources certainly cover a large enough share of news consumers/readers (as well as their reactions and comments to the news) to be able to detect the main characteristics of the phenomenon. So this sampling strategy enables us to investigate the general perception of our topics of interest, which concern two target groups of hate speech: migrants and members of the LGBT+ community. These target groups are the focus of the larger research project of which the present contribution is part (see also the discussion in Section Discussion). For the present contribution, however, both target groups are merged. For each of the Facebook pages, we identified posts (i.e., news articles re-posted by the media outlets) discussing these two topics/target groups. We selected the posts through (a) a keyword-based search and (b) a machine-learning classifier trained on already identified relevant posts, in order to find additional relevant posts. Finally, after these automated searches, we manually filtered the output (i.e., selected relevant posts).

**Table 1:** Selected mainstream media outlets and their Facebook pages.

**Language**	**News outlet**	**Facebook page**
English	BBC News	www.facebook.com/bbcnews
English	Daily Mail	www.facebook.com/DailyMail
English	The Guardian	www.facebook.com/theguardian
Dutch	Het Laatste Nieuws	www.facebook.com/hln.be
Dutch	Nieuwsblad	www.facebook.com/nieuwsblad.be
Dutch	VRT	www.facebook.com/vrt.be
Slovenian	24 ur	www.facebook.com/24urcom
Slovenian	Nova24TV	www.facebook.com/Nova24TV
Slovenian	Novice siol.net	www.facebook.com/SiOL.net.Novice
Croatian	24sata	www.facebook.com/24sata
Croatian	Index	www.facebook.com/index.hr
Croatian	Jutarnji list	www.facebook.com/jutarnji.list

After this topic-based selection of the media content, one final step was conducted: annotation.^3^ Each comment posted as a reaction on these Facebook posts was manually annotated for the phenomenon of hate speech by multiple trained, independent annotators. The annotators were (paid) students from the different universities that are involved in the research project, and one PhD candidate for Dutch (see below). They received proper training, guidelines, and support with respect to the task at hand. The annotation was performed in-context: annotators first read entire comment threads and then labeled each comment. They had to decide whether a comment was either acceptable speech or hate speech. They were instructed to include various types of hatefulness in the “hate speech” label, such as: inappropriate speech not aimed at someone (such as swearing or cursing), offensive speech aimed at someone, either for their background or not, and violence-inciting speech aimed at someone, either for their background or not. People's “background” includes for instance their religion, gender identity, sexual orientation, their nation, race, ethnicity, language, disability and potential refugee or migrant status. In the present study, we collapse this multi-level variable into a binary one, only distinguishing acceptable or non-hateful speech from hate speech (i.e., including all remaining categories). A more fine-grained analysis of hate speech, using all of the different subcategories that were annotated, is left for future work (see also the concluding Section Discussion).

Each of the comments in the dataset was annotated by multiple annotators for the fine-grained types of hate speech described above. For the binary hate speech class used in this work (hate speech vs. not hate speech), the achieved inter-annotator agreement is moderate (according to Landis and Koch, 1977 for a closely related metric). These agreement scores were calculated per language as Krippendorff's *alpha* (Krippendorff, 2018) and are summarized in Table 2. Note that a low inter-annotator agreement is common for hate speech detection and related tasks (such as toxic and abusive language detection) due to their subjective nature (Waseem, 2016). For English, Slovenian, and Croatian, there was one annotation round including eight independent annotators (students). Dutch was annotated at a later stage in the project. Based on the sufficiently high inter annotator agreement observed in the first three languages, we decided to include only two “regular” annotators for Dutch (students) and a highly skilled “super-annotator” (PhD candidate in linguistics) who resolved potential disagreements. As a final label, we used the mode (most frequently assigned category) of the different annotators' decisions for English, Slovenian, and Croatian, and the super-annotator's decisions for Dutch.

**Table 2:** Initial inter-annotator agreement scores.

**Language**	**Krippendorff's alpha**
English	0.409
Dutch	0.468
Slovenian	0.528
Croatian	0.520

### 3.2. Sociodemographic variables

In addition to the hate speech annotation of the comments in the corpus, the comment authors' sociodemographic profiles were manually annotated as well. Given the vast size of the initially collected corpus, it was only feasible to perform these manual annotations for a randomly selected subset of messages. In the remainder of this paper, and in all the analyses, the presented corpus refers to this annotated subset, and thus includes the relevant metadata on all of its authors. Three sociodemographic variables are included in the present research design: the language area of the users (operationalized as the language area of the media outlets–see above), their age, and their gender identity. These last two variables were annotated on a user-level through manual inspection of the users' Facebook profiles. Note that certain users' age and/or gender identity could not be identified with sufficient certainty. These users were excluded from the final corpus.

The users' language (area) is a four-level categorical variable, with the following labels: (British) English, (Flemish) Dutch, Slovenian, and Croatian. As for their gender identity, one of two labels (male/female) was assigned to users by the annotators, based on the available meta-information on Facebook. For age, finally, we work with a four-level categorical variable, distinguishing users under 25 years old, between 26 and 35 years old, between 36 and 65 years old, and finally older than 65. We opted for these specific four categories because for many people, they correspond to the following phases in life: “education years,” i.e., youth until the end of formal education or training (0–25), then the “working years,” divided in young adulthood (26–35) and adulthood (36–65), and finally retirement (65+).

The distribution of the Facebook comments in the corpus in terms of the news outlet's language and the author's gender identity and age is shown in Table 3. There is a notable difference in sample size concerning authors' gender (with more male than female data in all languages), age (with the least data for the youngest and oldest groups in all languages), and language (with the least data for English and the most for Dutch). However, we argue that our methodology is sufficiently robust to deal with this, as generalized linear models can handle data imbalances concerning different predictor levels well (see below).

**Table 3:** Distribution of hate speech (HS) and non-hate speech (non-HS) comments per language (area), gender identity, and age.

**Language**	**# Messages**	**Hate speech**	**Gender**		**Age**		
			**Male**	**Female**	**0–25**	**26–35**	**36–65**	**65**+
English	1,597	HS 619	445	174	34	229	292	64
		Non-HS 978	584	394	98	346	473	61
Dutch	4,703	HS 2,572	1,780	792	342	516	1,388	326
		Non-HS 2,131	1,144	987	502	432	1,010	187
Slovenian	2,099	HS 989	684	305	52	245	573	119
		Non-HS 1,110	645	465	85	355	540	130
Croatian	1,957	HS 1,126	791	335	142	236	708	40
		Non-HS 831	514	317	184	193	424	30

### 3.3. Method

In the analyses below (Section Results), we statistically model the probability of a Facebook comment (to a re-posted news article) being hateful based on the author's sociodemographic profile. We use binary generalized linear models (i.e., with a binomial distribution), which are the recommended and most straightforward models to analyze binary data. We use the model implementation from the R package “stats” (R Core Team, 2022). Each datapoint in the corpus represents a comment that was annotated as either hateful or non-hateful, which is the binary response for the models. We will investigate the impact on this response of three predictors (or fixed effects), i.e., three aspects of the authors' sociodemographic profiles: their age, gender identity, and language area (of the news outlet). Potential interactions between these predictors will be examined too.

Note that while we have access to author profile information per social media message, we could not obtain unique author identifiers for both a practical and an ethical reason: the required unique author information could not be scraped from Facebook with the software that we used, as this procedure goes against GDPR regulations. Consequently, we do not know which messages may have been written by the same author. For the models, this implies that we cannot include a random effect for author/subject in order to correct for potential repeated measurements. However, based on qualitative examination of this dataset as well as of comparable corpora, we assume the actual number of repeated measurements (i.e., multiple reactions by the same user) to be relatively small. The qualitative examination consisted in the manual checking of several comment threads below multiple Facebook posts (posted by the selected news outlets) from the dataset, in which we did not observe many obvious repetitive users based on usernames. While this is obviously a small-scale analysis of the data in this respect — as a pragmatic result of both privacy regulations and the vast size of the corpus —, it did give us an indication on the general practice of readers replying to news outlets, including its (absence of problematic) repeat reactions.

In the results section below, we discuss the models that best fit the data. This was experimentally determined through backward stepwise selection: the systematic deletion of insignificant predictors, determined with anova tests. We started from a full model including all potential interactions between the sociodemographic predictors, i.e., the two-way interaction between age and gender for the per-language models, and the three-way interaction between age, gender, and language, for the model for the entire dataset.

## 4. Results

Below, we discuss the best model for each of the four language-based subsets of the dataset: the English, Dutch, Slovenian, and Croatian subsets (Sections English to Croatian). Finally, we present a model for the entire dataset that includes language as a predictor (Section All).

### 4.1. English

The first subset of the data that we will zoom in on, consists of reader comments to Facebook posts by British English news outlets. These comments' (non-)hatefulness is best predicted by the authors' gender identity and age, but not by the interaction of these two variables–so the gender effect does not depend on the authors' age or vice versa. Table 4 shows the model's summary table.^4^ In the English dataset, men appear significantly more likely to produce hateful Facebook comments than women. This corroborates previous findings (see Section Theoretical framework) on gender divides in the production of hate speech (Waseem and Hovy, 2016; De Smedt et al., 2018), which in turn may be related to different attitudes on and a different degree of sensitivity to offensive speech by men and women (Lambe, 2004). Finally, the less frequent production of hate speech by female users might also relate to the repeatedly attested finding that women tend to write and speak in more polite or careful ways than men (e.g., including more hedges such as *I think, I guess*, ...), thus mitigating their opinion more (Newman et al., 2008).

**Table 4:** English subset: summary table.

	**Estimate**	**Std. Error**	**Z value**	**Pr (>|*z*|)**	**Sig**.
(Intercept)	−1.5449	0.2204	−7.010	2.38e-12	***
Age 26–35	0.7444	0.2187	3.404	0.000664	***
Age 36–65	0.6424	0.2141	3.000	0.002701	**
Age 65+	1.2797	0.2721	4.703	2.57e-06	***
Gender male	0.6136	0.1126	5.451	5.01e-08	***

Sig. codes: 0 “^***^” 0.001 “^**^” 0.01 “^*^” 0.05 “.” 0.1 “” 1.

**Figure 1:**
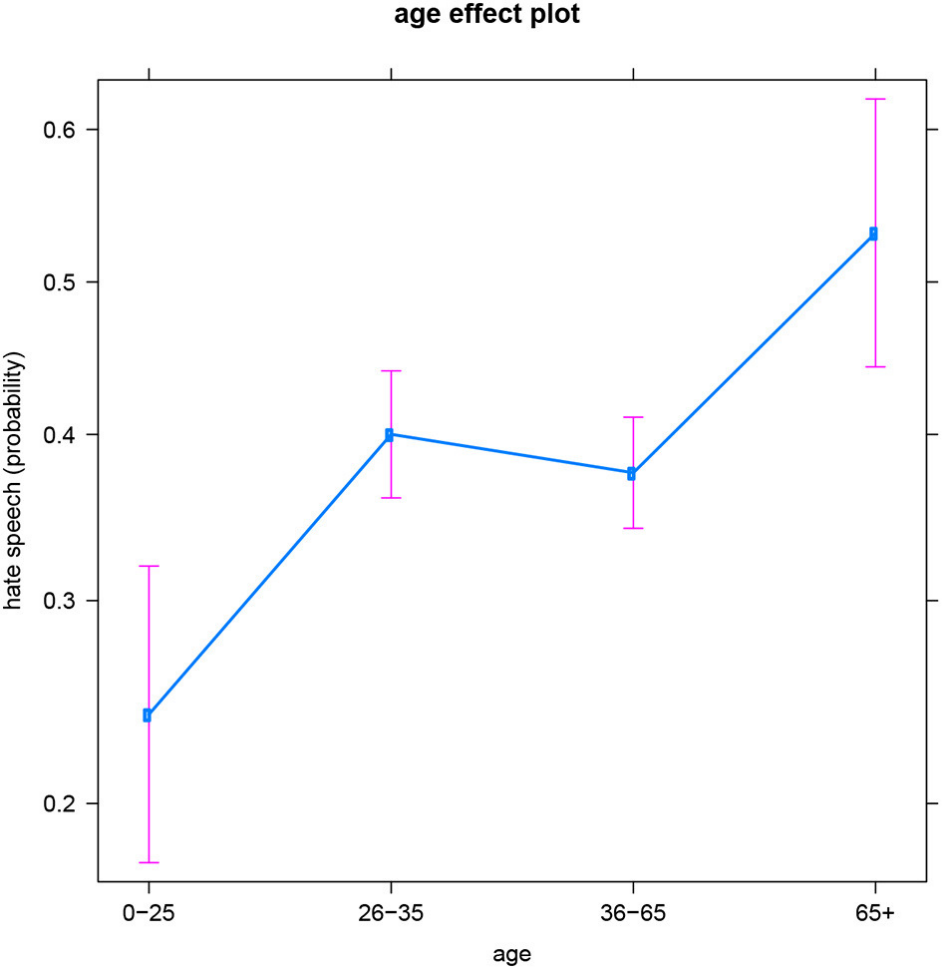
English subset: Effect of age on hate speech (predicted probabilities).

The general age trend in the English data is that the likelihood to produce hateful messages increases with age. This seems to confirm the popular belief that youths are more tolerant, but it also contradicts previous work in which young adults (aged 20–35) appeared most inclined to utter (jihadist) hate speech, i.e., more than older adults (De Smedt et al., 2018). While the specific topic or target of hate speech might play a role in these age patterns (e.g., an age-related “preference” for hate speech directed toward migrants and the LGBT+ community vs. jihadist hate speech), this falls outside the scope of the present contribution–but we come back to it in the Discussion. Recall that in the analyses, we distinguish between four consecutive age groups. In the English subset, each of these groups is significantly different from the others, except for the two middle groups: users between 26 and 35 years old vs. users between 36 and 65 years old (see also Figure 1^5^). So for English, we could regroup the four-level age variable into three levels instead: youths (0–25), young and middle aged adults (26–65), and older adults (65+). In the age period between youth and older age (i.e., 26–65), people's production of hateful online comments does not significantly increase or decrease. Note that the absence of a significant difference between these two age groups is not likely the result of a difference in sample size: recall that these two middle age categories are actually the best represented in all four languages and thus offer the models the most information and certainty (resulting in the plot in the smallest confidence intervals).

### 4.2. Dutch

**Figure 2:**
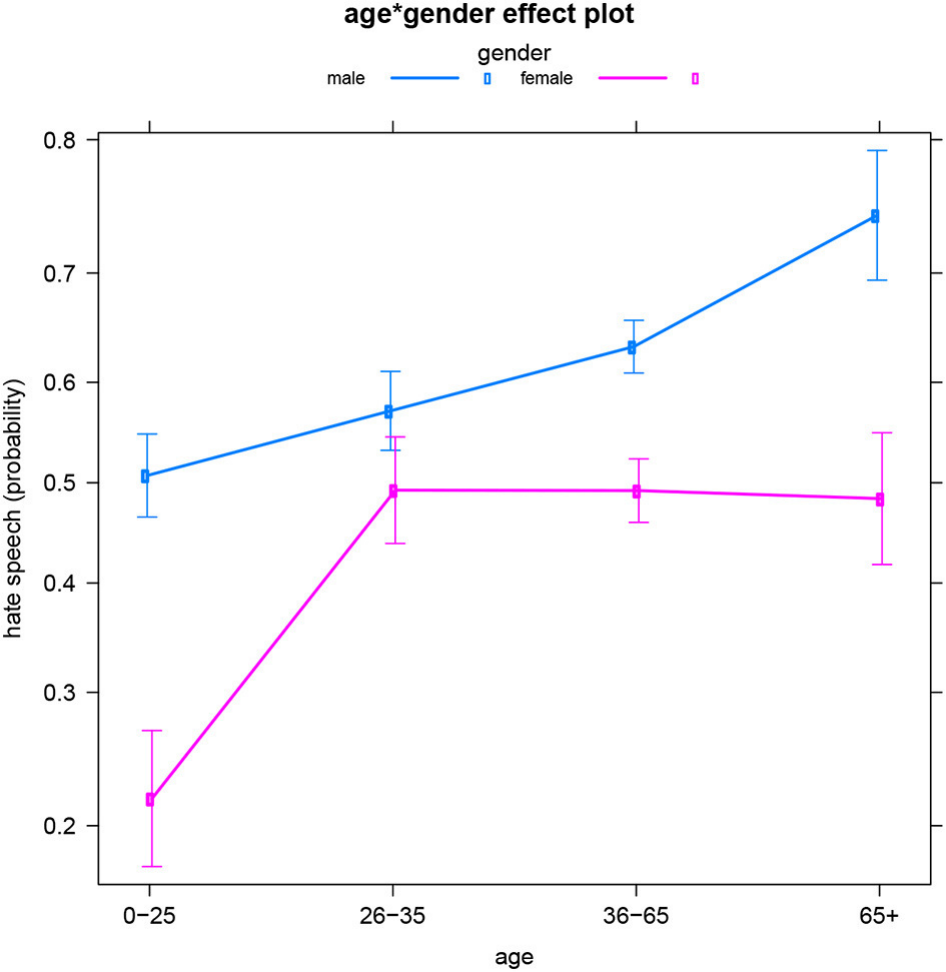
Dutch subset: Effect of age*gender on hate speech (predicted probabilities).

The next subset of the data concerns reader comments to Facebook posts from Flemish Dutch news outlets. In this subset, the comments' (non-)hatefulness is best predicted by the interaction between the authors' gender identity and age (see Table 5 for the summary table). Figure 2 illustrates the probability of female vs. male authors writing hateful posts depending on the authors' age, and vice versa. An overall gender pattern is that at any age, men are more likely to produce hate speech than (same-aged) women, which echoes our findings for English as well as previous work (see above). This gender divide is statistically significant in every age group, except for users aged 26–35.

**Table 5:** Dutch subset: summary table.

	**Estimate**	**Std. Error**	**Z value**	**Pr(>|*z*|)**	**Sig**.
(Intercept)	−1.2767	0.1403	−9.101	< 2e-16	***
Age 26–35	1.2464	0.1782	6.994	2.68e-12	***
Age 36–65	1.2445	0.1548	8.039	9.05e-16	***
Age 65+	1.2121	0.1953	6.207	5.38e-10	***
Gender male	1.3060	0.1643	7.947	1.91e-15	***
Age 26–35:Gender male	−0.9852	0.2138	−4.608	4.06e-06	***
Age 36–65:Gender male	−0.7238	0.1850	−3.912	9.15e-05	***
Age 65+:Gender male	−0.1607	0.2516	−0.639	0.523	

Sig. codes: 0 “^***^” 0.001 “^**^” 0.01 “^*^” 0.05 “.” 0.1 “” 1.

A general age trend is again that both men and women produce more hateful comments at older ages. However, Figure 2 reveals quite different specific age dynamics for men and women. The interaction pattern between age and gender is the following: while men's production of hate speech gradually increases as they age, women appear to reach some sort of “hate plateau” between the age of 26 and 35, i.e., they do not continue to post more hate comments after the age of 35. In order to explain this pattern, sociological research is required (see also the concluding Section Discussion).

Finally, note how this interaction is quite different from the observed pattern for the English data (with the age pattern not differing for men and women), which suggests a sociocultural difference between the English-speaking and Dutch-speaking areas. We will come back to this later.

### 4.3. Slovenian

The hatefulness of reader comments to Slovenian news outlets' Facebook posts is best predicted by the authors' gender identity and age, but not by the interaction of these two variables (see Table 6 for the summary table). In this Slovenian subset of the data, men are once again significantly more likely to write hateful messages than women, just like in the English and Dutch data.

**Table 6:** Slovenian subset: summary table.

	**Estimate**	**Std. Error**	**Z value**	**Pr(>|*z*|)**	**Sig**.
(Intercept)	−0.75755	0.18554	−4.083	4.45e-05	***
Age 26–35	0.10802	0.19583	0.552	0.58123	***
Age 36–65	0.51033	0.18722	2.726	0.00641	**
Age 65+	0.40571	0.21832	1.858	0.06313	.
Gender male	0.45624	0.09255	4.930	8.23e-07	***

Sig. codes: 0 “^***^” 0.001 “^**^” 0.01 “^*^” 0.05 “.” 0.1 “” 1.

**Figure 3:**
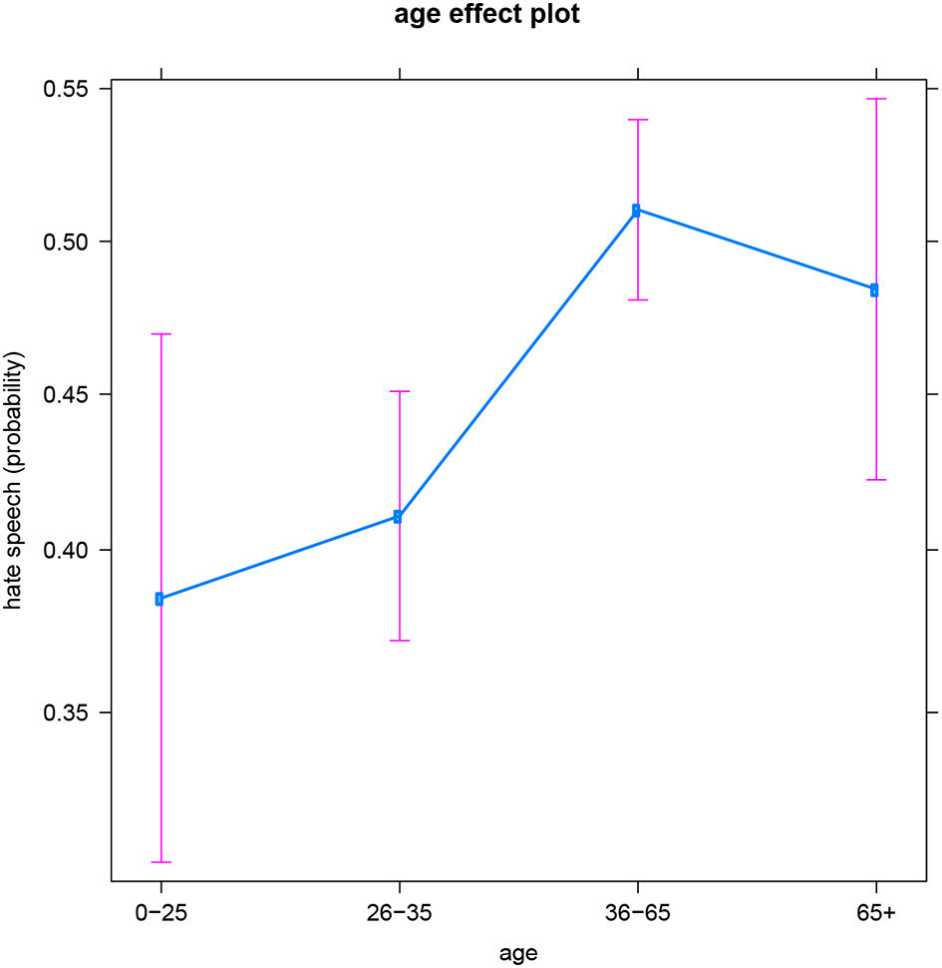
Slovenian subset: Effect of age on hate speech (predicted probabilities).

The general age trend is similar too, with more hate posts being produced at older ages (both by men and women). However, the two youngest groups (users aged 0–25 and 26–35) are not significantly different from each other, and neither are the two oldest groups (users aged 36–65 and 65+). This is visualized by Figure 3. So for Facebook users in the Slovenian dataset, it seems more sensible to work with a binary age variable comparing people under 35 to people over 35. Consequently, the age of 35 appears to be some sort of tipping point in Slovenian people's production of hateful online comments. This differs from the specific age patterns found in the English and Dutch datasets, and points toward a cultural difference between these three language areas (see below for a more elaborate discussion). But recall also that the youngest and oldest age groups are the least represented in the dataset, and thus offer the model the least information and certainty to draw conclusions (resulting in the larger confidence intervals on the plot). Consequently, the collection of more material for these age groups might be insightful. We come back to this in the Discussion.

### 4.4 Croatian

The final subset of the data concerns reader comments on Facebook pages of popular Croatian news outlets. These comments' hatefulness is best predicted by the authors' gender identity and age, but not by their interaction (see Table 7 for the summary table). Just like for the other three languages, and as attested in previous work (see above), men in the Croatian dataset are significantly more likely to produce hateful messages than women.

**Table 7:** Croatian subset: summary table.

	**Estimate**	**Std. Error**	**Z value**	**Pr(>|*z*|)**	**Sig**.
(Intercept)	−0.48150	0.12874	−3.740	0.000184	***
Age 26–35	0.47112	0.14854	3.172	0.001516	**
Age 36–65	0.75369	0.12794	5.891	3.84e-09	***
Age 65+	0.56785	0.26715	2.126	0.033536	*
Gender male	0.34707	0.09789	3.545	0.000392	***

Sig. codes: 0 “^***^” 0.001 “^**^” 0.01 “^*^” 0.05 “.” 0.1 “” 1.

**Figure 4:**
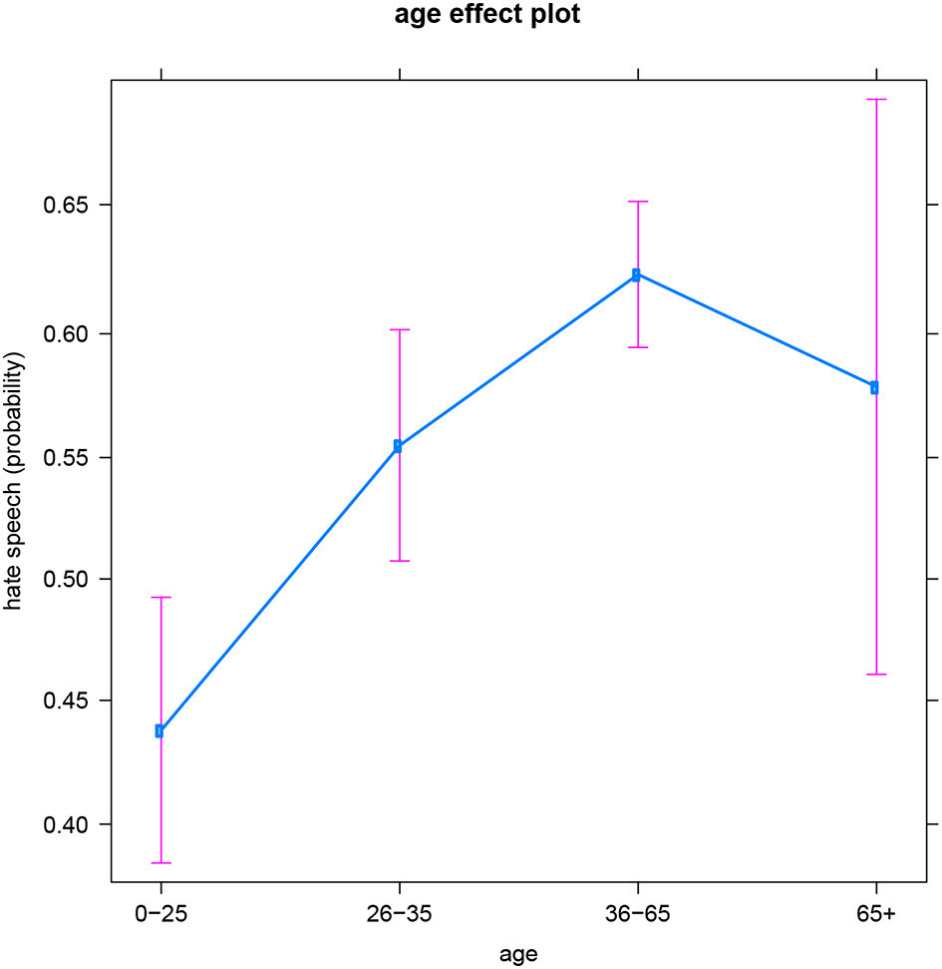
Croatian subset: Effect of age on hate speech (predicted probabilities).

With respect to the general age pattern, once again more hate comments seem to be produced at older ages (both by men and women). However, the oldest group (people over 65) seems to be an exception, displaying much variation in hatefulness among individuals—this is illustrated by the large confidence interval for this group in Figure 4 (which might also relate to the smaller sample size for this group—see the Discussion). Consequently, this age group is not significantly different from any other group. So while the probability of producing hateful online messages significantly increases from youth (0–25) to younger adulthood (26–35) and then marginally insignificantly increases to later adulthood (36–65), none of these three groups significantly differ (regarding the production of hate speech) from people over 65. So, unlike for the other language-based subsets of the data, the results for Croatian suggest that another (e.g., social, political, cultural,…) variable is at play for the older group, accounting for the large variability within this age category–see also the Discussion.

### 4.5. All

The separate models for each of the four language-based subsets of the data (Sections English to Croatian) have revealed both similarities and differences between the subcorpora with respect to the profiles of hate speech authors. In this section, we will analyze the entire dataset, including all four languages, and include language as a predictor in the model. This will enable a systematic and statistical comparison between the languages, as well as a verification of which language-specific tendencies significantly differ from each other and which ones do not.

**Figure 5:**
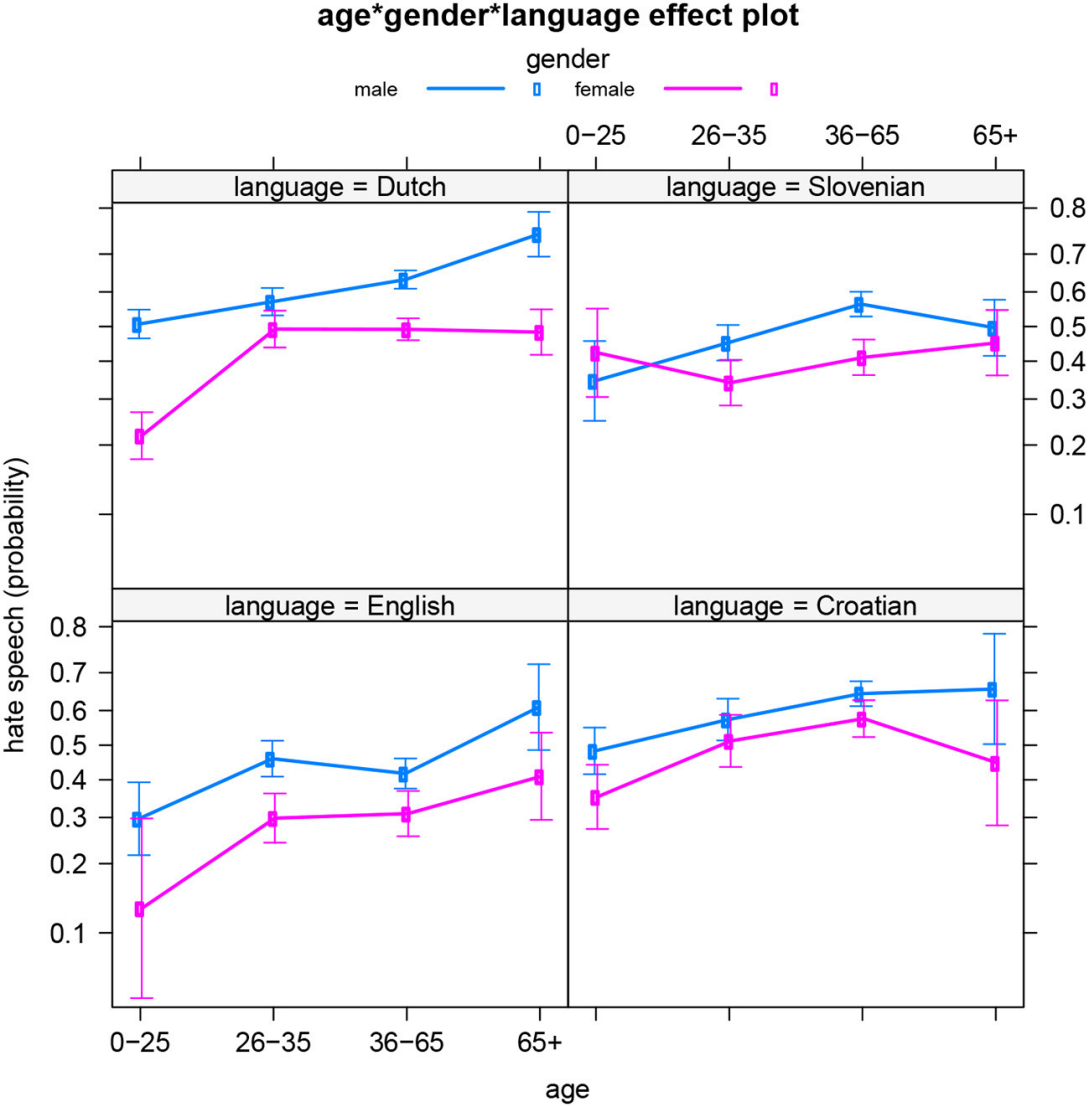
All languages: Effect of age*gender*language on hate speech (predicted probabilities).

In the best fitting model for the data, a three-way interaction is included between the three sociodemographic variables: authors' gender identity, their age, and the language (area) of the news outlet page. This three-way interaction significantly predicts the hatefulness of the reader comments (Table 8 presents the summary table). Figure 5 illustrates the interaction by showing the age and gender patterns per language. As observed in the separate models above, Dutch stands out, with clearly different age dynamics for men and women. And for Slovenian, we now see that the highs and lows in male and female hate speech occur at different ages, but that both gender groups do not significantly differ from each other regarding hateful writing in the youngest and oldest age groups. This was not yet revealed by the separate model for Slovenian language. So the current dataset indicates that Slovenian men and women express their feelings of hatred and anger (and related emotions) to the highest extent at different points in their lives. It also suggests that men and women's social realities may differ in Slovenia between the working/active years of 26 and 65, but not so much during youth (−26) and older age (65+).

**Table 8:** All languages: summary table.

	**Estimate**	**Std. Error**	**Z value**	**Pr(>|*z*|)**	**Sig**.
(Intercept)	−1.90954	0.53576	−3.564	0.000365	***
Age 26–35	1.05577	0.55555	1.900	0.057381	.
Age 36–65	1.10851	0.55261	2.006	0.044861	*
Age 65+	1.54490	0.59566	2.594	0.009498	**
Gender male	1.04806	0.57832	1.812	0.069948	.
Language Croatian	1.30341	0.56906	2.290	0.021996	*
Language Dutch	0.63289	0.55382	1.143	0.253129	
Language Slovenian	1.60206	0.59703	2.683	0.007288	**
Age 26–35:Gender male	−0.35281	0.60616	−0.582	0.560534	
Age 36–65:Gender male	−0.57946	0.60071	−0.965	0.334731	
Age 65+:Gender male	−0.23873	0.68401	−0.349	0.727077	
Age 26–35:Language Croatian	−0.40143	0.60790	−0.660	0.509023	
Age 36–65:Language Croatian	−0.19222	0.59523	0.323	0.746749	
Age 65+:Language Croatian	−1.14640	0.72872	−1.573	0.115679	
Age 26–35:Language Dutch	0.19067	0.58344	0.327	0.743823	
Age 36–65:Language Dutch	0.13599	0.57388	0.237	0.812687	
Age 65+:Language Dutch	−0.33279	0.62685	−0.531	0.595498	
Age 26–35:Language Slovenian	−1.40416	0.62974	−2.230	0.025764	*
Age 36–65:Language Slovenian	−1.16102	0.62137	−1.868	0.061695	.
Age 65+:Language Slovenian	−1.42666	0.67993	−2.098	0.035883	*
Gender male: Language Croatian	−0.50958	0.62498	−0.815	0.414865	
Gender male: Language Dutch	0.25790	0.60121	0.429	0.667951	
Gender male: Language Slovenian	−1.37656	0.67861	−2.029	0.042508	*
Age 26–35:Gender male: Language Croatian	0.06491	0.68061	0.095	0.924015	
Age 36–65:Gender male: Language Croatian	0.33275	0.65926	0.505	0.613745	
Age 65+:Gender male: Language Croatian	0.56467	0.87858	0.643	0.520412	
Age 26–35:Gender male: Language Dutch	−0.63241	0.64276	−0.984	0.325164	
Age 36–65:Gender male: Language Dutch	−0.14436	0.62856	−0.230	0.818348	
Age 65+:Gender male: Language Dutch	0.07799	0.72883	0.107	0.914786	
Age 26–35:Gender male: Language Slovenian	1.14774	0.72334	1.587	0.112575	
Age 36–65:Gender male: Language Slovenian	1.53152	0.70970	2.158	0.030929	*
Age 65+:Gender male: Language Slovenian	0.74249	0.81239	0.914	0.360738	

Sig. codes: 0 “^***^” 0.001 “^**^” 0.01 “^*^” 0.05 “.” 0.1 “” 1.

Note that apart from this additional finding for Slovenian, the present model does not just corroborate the previous models' results. It also confirms that the differences between the languages are statistically significant. Finally, we ran four additional models in which we compared each language to all three other languages combined. These models (which we will not discuss in detail) confirmed the same previously observed trends.

## 5. Discussion

The importance of and interest in the identification of online hateful content has increased considerably these last years. This has led to the development of a variety of approaches in the field of natural language processing (NLP) that aim to automatically flag this type of content (Mandl et al., 2019; Zampieri et al., 2019, 2020). Previous work has shown the importance of the inclusion of author demographics in the study of hate speech (see Section Theoretical framework), as it can contribute to the development of strategies that counter hateful discourse, as well as to more robust, less biased and better performing classification models.

The present paper aimed to explore the profiles of hate speech authors in a multilingual dataset (including English, Dutch, Slovenian, and Croatian) of readers' comments to news outlets' Facebook posts concerning migrants or the LGBT+ community. We focused on the sociodemographic variables of age and gender identity in particular, in interaction with each other and with users' language (area) or culture. Our analyses reveal both similarities and differences between the four language-based subsets of the dataset regarding the profiles of hate speech authors. In all four languages, men appear more likely than women to produce online hate comments (as a reaction to media outlets' Facebook posts), and people appear to produce more hate speech as they grow older. These two trends confirm findings from previous work (see Section Theoretical framework). The more detailed age patterns, however, add important nuance, as they show that these commonly observed trends do vary slightly in different languages or language areas. For English, it appears ideal to approach author age–regarding its impact on the production of hateful Facebook comments–as a categorical variable with three levels: 0–25 years old (largely corresponding to youths until the end of formal education/training) vs. 26–65 years old (active years) vs. 65+ (retirement). But for Slovenian, a binary age classification seems preferable (0–35 years old vs. 35+). And in Croatian, the eldest group (65+) is an outlier with much variation regarding hate speech production, and does not differ significantly from any other age group. Finally, Dutch stands out because the observed age pattern differs for men and women: men continue to produce more hate speech as they grow older, whereas women reach a sort of “hate plateau” between the age of 26 and 35. These differences between the four subsets of the data suggest that distinct social, cultural, and/or political realities might be at play in these respective language areas. In fact, the sociocultural context of data collection differed to some extent for the respective language areas and communities. Since the research project started with a Slovenian focus, the news topics for the dataset were selected based on two phenomena that were in progress in Slovenia at the time of collection: (a) an unprecedented migrant crisis (the so-called “Balkan route”), and (b) a referendum campaign on LGBT+ rights. At that time, similar contexts and situations occurred in Croatia too–(a) a migrants crisis of similar proportions and (b) a “marriage referendum” defining marriage as a community of man and woman–but not in Belgium or in the UK, especially on the LGBT+ front. So the collected news posts and their reader comments were more affected by ongoing events for Slovenian and Croatian, and were somewhat more “general” for Dutch and English, especially for the LGBT+ topic. It is probable that topics that are more current, real-time, and local, evoke hateful reactions to a different extent than more general, global subjects. So the specific type of hate speech that is under investigation (with respect to targeted groups) may play a role and should be taken into consideration when interpreting the findings, in tandem with the regions and cultures from which the data are derived. Finally, the plots showed how for Slovenian and Croatian only, the production of hateful messages went down for the eldest group (65+) (although not always significantly so, due to the higher variation in this age group). An additional factor that may be at play for older people in Croatia and Slovenia, but not in Belgium or the UK, is having lived under a socialistic regime. This might (in part) explain the lower probability of hate speech among older people for these language areas: it could be related to a less outspoken inclination to openly express opinions in general. In addition, former Yugoslavia's active promotion of multiculturality may play a role too (Kuhar and Ceplak, 2016). But further sociological and sociohistorical research is required to inspect these hypotheses.

Follow-up research could not only explore the position of older adults (65+) in Croatian and Slovenian society, but also zoom in on potential life changes that Slovenians face around the age of 35, and examine how female and male realities may diverge between the age of 26 and 35 in Flanders. In addition, capturing users' age in a more fine-grained way, e.g., by including more categories in the annotations or by using exact age or birth year, could yield more detailed age patterns. And so could the collection of additional material for the least represented age categories, and/or a resampling resulting in more even distributions concerning user metadata. However, note that the uneven distribution in the corpus regarding e.g. users' age is not random or coincidental, but is informative in itself, reflecting actual user distributions on social media platforms such as Facebook. Another follow-up analysis concerns a more fine-grained approach to hate speech, in which the different subcategories of hateful discourse that were annotated are distinguished, as well as the two different target groups that were now merged, since people's inclination to post hateful reactions online may not manifest in the same way across different topics. And topics could interact with people's profiles or regions too: for instance, certain topics may elicit more hate from a certain gender or age group in a certain region, because of cultural and contextual differences. Other paths for future work consist in analyzing different aspects of authors' sociodemographic profiles, such as their social class or level of education. Properties with respect to social networks could be inspected too. It could for instance be investigated whether the size and nature of a Facebook user's online network, or their activity on social media, influences their production of hateful posts. And it may be interesting to compare the present results to findings for other languages, and verify whether the (general) age and gender trends indeed hold, and which potential differences and nuances emerge. In terms of generalizing our findings, reproducing this paper's experiments after gathering additional data (especially for the languages and age groups that are less well-represented) can strengthen or nuance our conclusions and increase statistical power. Finally, an interesting future line of research would be how particular unusual circumstances and crises such as pandemics, refugee crises, and environmental crises may influence the hate speech landscape, including the profiles of prototypical perpetrators.

In conclusion, our results corroborate previous findings on the age and gender identity of prototypical online “haters,” while also adding important nuance and showing that specific age and gender dynamics differ in different language areas (which also correspond to different regions, societies, and cultures)–even when these are not that far apart (recall that all four selected language areas belong to countries in Europe). The fine-grained age and gender profiles of hateful content creators that our analyses reveal, can be used as information (e.g., as features) in future hate speech detection tasks, as well as for sensibilization on and counter-initiatives to the spread of (online) hatred.

## Data availability statement

The datasets presented in this study can be found in online repositories. The names of the repository/repositories and accession number(s) can be found below: http://hdl.handle.net/11356/1483.

## Author contributions

All authors listed have made a substantial, direct, and intellectual contribution to the work and approved it for publication.

## Footnotes

^1^For English, only British media outlets were selected since we aim to investigate online hate speech in a European context. For Dutch, only Flemish media outlets (i.e., from Flanders, northern Belgium) are included due to the scope of the underlying project.

^2^ https://www.alexa.com/topsites/countries

^3^For detailed information on the annotation procedure, see Ljubešić et al. (2019). The annotation guidelines for the project can be consulted here: http://hdl.handle.net/11356/1462

^4^The summary tables below show the impact of the different predictors per model. For all predictor levels, summary statistics are provided in comparison to the reference group: here always the youngest group of female authors (0–25). The “Estimate” expresses how the predictor level affects the predicted probability of hateful writing. This coefficient is accompanied by a standard error indicating its spread, and a z- and p-value indicating its significance (compared to the reference category).

^5^Recall that these binary models predict the probability (expressed between 0 and 1) of an author producing a hateful message, given aspects of that author's sociodemographic profile. For instance, Figure 1 shows that people younger than 25 have a 24% chance of producing online hate speech in this dataset. The intervals on the plot are the 95% confidence intervals.
